# Early adversity and inflammation at midlife: the moderating role of internalizing psychopathology

**DOI:** 10.1017/S0033291724002265

**Published:** 2025-01-09

**Authors:** Zachary S. Michal, Craig A. Marquardt, Robert F. Krueger, Paul A. Arbisi, Noah C. Venables

**Affiliations:** 1 Minneapolis Veterans Affairs Health Care System;; 2 Department of Psychiatry and Behavioral Sciences, University of Minnesota;; 3 Department of Psychology, University of Minnesota-Twin Cities; 4 Minnesota Psychological Services, LLC, Maple Grove, MN, USA

**Keywords:** childhood adversity, inflammation, internalizing psychopathology, neuroticism

## Abstract

**Background.:**

Childhood adversity has been associated with increased peripheral inflammation in adulthood. However, not all individuals who experience early adversity develop these inflammatory outcomes. Separately, there is also a link between various internalizing emotional distress conditions (e.g. depression, anxiety, and fear) and inflammation in adulthood. It is possible the combination of adult emotional distress and past childhood adversity may be uniquely important for explaining psychopathology-related immune dysfunction at midlife.

**Methods.:**

Using data from the Midlife in the United States (MIDUS) study (*n* = 1255), we examined whether internalizing, defined as past 12-month emotional distress symptomatology and trait neuroticism, moderated associations between childhood adversity and heightened inflammation in adulthood. Using latent variable modeling, we examined whether transdiagnostic or disorder-specific features of emotional distress better predicted inflammation.

**Results.:**

We observed that childhood adversity only predicted adult inflammation when participants also reported adult internalizing emotional distress. Furthermore, this moderation effect was specific to the transdiagnostic factor of emotional distress rather than the disorder-specific features.

**Conclusions.:**

We discuss the possibility that adult internalizing symptoms and trait neuroticism together may signal the presence of temporally stable vulnerabilities that amplify the impact of childhood adversity on midlife immune alterations. The study highlights the importance of identifying emotional distress in individuals who have experienced childhood adversity to address long-term immune outcomes and enhance overall health.

Inflammatory markers encompass a variety of proteins and signaling molecules that facilitate immune activity and maintain overall health. Concentrations of inflammatory markers measured in the blood are often used to assess immune functioning. Chronic inflammation, marked by a persistent state of heightened immune activity, is associated with the development of conditions including heart disease and type 2 diabetes (Danesh et al., 2008; Theuma & Fonseca, 2003). Psychosocial stressors can activate inflammatory responses with implications for a variety of health outcomes (Liu, Wang, & Jiang, 2017; Steptoe, Hamer, & Chida, 2007). Childhood adversity in particular is an established environmental risk factor for the health conditions mentioned above and chronic inflammation later in life (Hostinar, Lachman, Mroczek, Seeman, & Miller, 2015; Kerr, McDonald, & Minnis, 2021; Lacey, Pinto Pereira, Li, & Danese, 2020). Thus, inflammation is likely a biological mechanism connecting childhood adversity to poor health during adulthood. However, not all individuals with childhood adversity exhibit later inflammation. Differences among individual vulnerabilities to stressors may play a role. Using a nationally representative sample of United States adults, we investigated whether the association between early adversity and adult inflammation is moderated by past 12-month emotional distress symptoms and trait neuroticism.

There is a reliable association between early life stress and increased health complications in adulthood (e.g. chronic inflammation); however, the severity and frequency of these outcomes can vary widely across individuals (Hostinar & Miller, 2019; Oh et al., 2018). Factors tied to stress vulnerabilities, including how individuals tend to appraise and respond to adversity, may modulate these health outcomes. For example, a recent study using data from the Midlife in the United States (MIDUS) project found that adults with a history of early adversity using more adaptive coping strategies experienced fewer physical health conditions, including auto-immune and bone conditions, compared to those using these strategies less often (Sheffler, Piazza, Quinn, Sachs-Ericsson, & Stanley, 2019). Similarly, in adults who experienced childhood maltreatment, higher self-reported control of emotions was associated with lower inflammation (Gouin, Caldwell, Woods, & Malarkey, 2017; Jones, Marsland, & Gianaros, 2023). Therefore, the long-term effects of early life stress may vary as a function of trait-level individual differences – one’s personal capacity to respond adaptively may influence the likelihood of chronic inflammation during adulthood.

Emotional distress psychopathology along with elevated personality traits of neuroticism reflect putative stress vulnerabilities capable of moderating the association between early adversity and later inflammation. Emotional distress conditions (e.g. generalized anxiety disorder [GAD] and major depressive disorder [MDD]) are more common among individuals who have experienced early adversity (Danese et al., 2008; Nanni, Uher, & Danese, 2012). Moreover, these conditions have been shown to be associated with reduced immune system functioning (Beurel, Toups, & Nemeroff, 2020). Despite this, symptoms of anxiety and depression, if included in statistical models of adversity and inflammation, are often treated as confounds (and modeled as covariates) rather than as potential moderators (Coelho, Viola, Walss-Bass, Brietzke, & Grassi-Oliveira, 2014). Conversely, in studies examining MDD and GAD as main effect statistical predictors of inflammatory markers (Costello, Gould, Abrol, & Howard, 2019; Duivis, Vogelzangs, Kupper, de Jonge, & Penninx, 2013; Michopoulos, Powers, Gillespie, Ressler, & Jovanovic, 2017; Ogłodek, Szota, Just, Szromek, & Araszkiewicz, 2016), the role of early adversity is not always examined. While some studies have examined the joint effects of emotional distress symptoms and early adversity on inflammation at midlife, inconsistencies exist. Specifically, some studies indicate that individuals with both depressive symptoms and early adversity have higher inflammation levels than those with only adversity but no depressive symptoms (Danese et al., 2008; Gill et al., 2020). However, other studies have found no differences in inflammatory markers when comparing these groups (Baumeister, Akhtar, Ciufolini, Pariante, & Mondelli, 2016; Coelho et al., 2014; Palmos et al., 2019).

Inconsistencies surrounding the combined influence of emotional distress symptoms and early adversity may stem in part from reliance on case-control methodology. Case-control designs group individuals based on discrete diagnostic categories such as MDD or GAD, which limits the ability to study how symptoms co-occurring across diagnoses may influence inflammatory outcomes (Moriarity & Alloy, 2020; Thylur & Goldsmith, 2022). *Transdiagnostic* internalizing (i.e. emotional distress) symptoms are psychiatric comorbidities commonly observed across depression, anxiety, and fear-based conditions (Krueger, 1999). These transdiagnostic features are often overlooked in case-control research, potentially obscuring when similar associations with inflammation exist across various internalizing conditions. Indeed, one meta-analysis found that diagnoses of clinical depression and anxiety both demonstrated similarly elevated inflammation compared to non-clinical populations (Tursich et al., 2014); this is consistent with a shared, transdiagnostic mechanism across these conditions.

Neuroticism is a broad personality trait associated with heightened emotional reactivity, tendencies towards negative affect, and increased sensitivity and reactivity to stressors (Claridge & Davis, 2001; Griffith et al., 2010). It is relatively stable during adulthood and acts as a non-specific predictor of various internalizing conditions (Costa & McCrae, 1988; Ormel & Wohlfarth, 1991). Importantly, measures of neuroticism assess content closely associated with the transdiagnostic comorbidities common across emotional distress diagnoses. In other words, neuroticism can serve as an effective marker of the shared variance across depression, anxiety, and fear-based psychopathologies (Griffith et al., 2010). Evidence for associations between neuroticism and chronic inflammation is mixed. An earlier meta-analysis, which included MIDUS data, did not observe a consistent main effect association between neuroticism and inflammation (Luchetti, Barkley, Stephan, Terracciano, & Sutin, 2014). Yet, the combined effect of neuroticism and adversity can be critical when considering associations with inflammation (Wang et al., 2022). Elliot, Turiano, and Chapman (2017) performed a moderation analysis within a subsample of MIDUS examining the personality trait of neuroticism combined with socioeconomic status (SES) to predict individual inflammation markers. Lower SES was associated with inflammation, but only when neuroticism was high. Given the known connection between lower SES and adverse childhood experiences (Walsh, McCartney, Smith, & Armour, 2019), it is possible that neuroticism specifically and transdiagnostic emotional distress more generally also reflect vulnerabilities to adverse life events on health outcomes.

The current study expands upon previous MIDUS research, which demonstrated associations between early adversity and individual markers of adult inflammation (Hostinar et al., 2015), as well as separate associations between transdiagnostic internalizing psychopathology and general health outcomes (e.g. all-cause mortality; Kim et al., 2021). Specifically, we investigated whether (Aim 1) the relationship between childhood adversity and inflammation at midlife is moderated by a latent estimate of past 12-month internalizing symptomatology and trait neuroticism using confirmatory factor analysis (CFA). In addition, we directly compared (Aim 2) transdiagnostic *v*. disorder-specific emotional distress (i.e. residual terms) when examining the connection between early adversity and inflammation in adulthood. Furthermore, our analyses used a latent estimate of chronic inflammation across multiple biomarker assays. This study is consistent with the goals of MIDUS to better understand the transdiagnostic processes of psychosocial resilience, adult well-being, and physical health. Ultimately, this may aid in identifying adults with childhood adversity who are vulnerable to worse health outcomes at midlife, thereby informing screening efforts focused on prevention and treatment.

## Methods

### Sample

This study used publicly available data from the MIDUS project (Ryff et al., 2007). In 1995, MIDUS recruited 7108 non-institutionalized adults via random digit dialing from the contiguous United States. In 2004, 5555 participants (75%) of the original sample were recruited for a second wave of data collection (MIDUS II). In the second wave, The Biomarker Project was initiated, involving a subsample (*n* = 1054) who underwent a 2-day study visit, including the collection of blood. Additionally, an African American subsample from MIDUS I was re- recruited and 201 of these individuals completed the Biomarkers protocol. In total, 1255 participants contributed valid biomarker data. The average time between the questionnaires/phone interviews and biomarker collection was 26.5 (s.d. = 14.0) months. For our analyses, 1.5% of the data was missing. Missing data was deemed completely at random using the vis_miss() visualization function within the R package Naniar (Tierney & Cook, 2020); multiple imputation was employed (Slopen et al., 2010). All data transformations were performed after reporting sample characteristics in Table 1.

## Measures

### Inflammation

Serum markers of inflammation were collected from fasting blood samples. C-Reactive Protein (CRP) was quantified using a particle-enhanced immunonephelometric assay (BNII nephelometer, Dade Behring Inc., IL). Interleukin-6 (IL-6) was measured with the Quantikine High-sensitivity ELISA kit #HS600B (R&D Systems, MN). Fibrinogen (FGN) assessment used the BNII nephelometer (N Antiserum to Human Fibrinogen; Dade Behring Inc., IL). E-Selectin was measured through a high-sensitivity ELISA assay (Parameter Human sE-Selectin Immunoassay; R&D Systems, MN). Soluble Intracellular Adhesion Molecule-1 (ICAM-1) was quantified with an ELISA assay (Parameter Human sICAM-1 Immunoassay; R&D Systems, MN). Intra- and inter-assay coefficients of variance were in acceptable range (<10%) for all assays. Guided by previous MIDUS analyses (Hostinar et al., 2015), values exceeding the 99.9th percentile were replaced with values 4 s.d.s from the mean. IL-6 and CRP were log-transformed to correct for positively skewed distributions (Friedman & Herd, 2010; Slopen et al., 2010; Straka, Tran, Millwood, Swanson, & Kuhlman, 2021). All inflammatory variables were *z*-transformed.

### Internalizing distress

Four continuous mental health measures were used to model a transdiagnostic internalizing distress factor (Kim et al., 2021). Past 12-month depression, anxiety, and panic were assessed using the Composite International Diagnostic Interview–Short Form (CIDI-SF) version 10. Thus, this assessment timeframe reflects more typical emotional functioning rather than acute states. The CIDI-SF has high agreement (91–94%) with its lengthier CIDI counterpart (Kessler, Andrews, Mroczek, Ustun, & Wittchen, 1998). The fourth indicator assessed neuroticism, which was developed from existing personality inventories and exhibits acceptable internal consistency (Cronbach’s *α* = 0.74) (Lachman & Weaver, 2007). Neuroticism reflects a general disposition towards negative emotions and is an effective transdiagnostic marker of internalizing distress (Griffith et al., 2010). Depression, anxiety, and panic measures were log-transformed following other MIDUS analyses (Eaton, Krueger, & Oltmanns, 2011). All internalizing measures were *z* transformed.

### Adverse childhood experiences

A cumulative sum of adverse childhood experiences (ACEs) (Felitti et al., 1998) was constructed consistent with previous MIDUS studies (Hostinar et al., 2015; Sheffler et al., 2019). MIDUS assessed eight historical experiences (before age 18): physical abuse, emotional abuse, sexual abuse, emotional neglect, physical neglect, parental divorce, parental substance abuse, and parental depression. The first five ACEs listed above were obtained from Childhood Trauma Questionnaire (CTQ) scales and collapsed into binary responses using thresholds listed in the manual (Bernstein & Fink, 1998). The CTQ is an established measure of childhood adversity demonstrates high agreement with external sources (Bernstein et al., 2003). The latter three ACEs items are specific to MIDUS II questionnaires (yes/no format) with questions like ‘Parent’s drug use ever caused problems?’ and ‘Mother depression?’ Only events occurring before age 18 were counted. Due to less frequent endorsement of 4 or more items as well as evidence that 4 or more ACEs is associated with worse clinical outcomes (Mersky, Topitzes, & Reynolds, 2013), total scores equal to or greater than 4 were collapsed into one category. Pre-imputation class memberships for ACEs levels were as follows: 0 (*n* = 583), 1 (*n* = 286), 2 (*n* = 132), 3 (*n* = 90), and ≽ 4 (*n* = 103).

### Covariates

Sociodemographic variables were included as covariates in follow-up analyses ([see Analyses] [https://midus.wisc.edu/] Ryff et al., 2007). Sex assigned at birth and marital status were dummy coded (0 = male, 1 = female; 0 = married, 1 = non-married). Following previous methods (Elliot & Chapman, 2016; Friedman & Herd, 2010), the most common race/ethnicity (white-non-Hispanic) was used as the reference, while less common African American, Hispanic, or other race/ethnicity identities were collapsed to 1. Household income reflected total family income in the past 12 months. Educational attainment ranged from 1 = no school/grade school to 12 = Ph.D./M.D./professional degree. Sociodemographic factors such as these have been associated with inflammation (Friedman & Herd, 2010; Nowakowski & Sumerau, 2015).

Furthermore, smoking status and past month alcohol consumption served as health covariates in follow-up analyses (see Analyses). Smoking status was dummy coded, with non-smokers as the baseline. Alcohol consumption was categorized as 1 = Never, 2 = Less than 1x/week, 3 = 1–2x/week, 4 = 3–4x/week, 5 = 5–6x/week, and 6 = Daily. Both smoking status and alcohol intake have been found to influence inflammation (Gonçalves et al., 2011; Imhof et al., 2001). Body mass index (BMI), exercise, and presence of medical conditions were also examined as health covariates due to their known associations with inflammation (Metsios, Moe, & Kitas, 2020; Pahwa, Goyal, & Jialal, 2023; Rodríguez-Hernández, Simental-Mendía, Rodríguez-Ramírez, & Reyes-Romero, 2013). Exercise was binary (1 = 20 min or more physical activity 3x/week). Various other chronic conditions were coded as 0 = No Condition and 1 = Present Condition.

## Analyses

All analyses were performed using RStudio version 1.4.1717 (R version 4.1.0). We performed exploratory factor analyses (EFA; ‘Psych’ package) with oblimin rotations to examine the factor structure of mental health and inflammation variables. We used scree plots and initial loading cutoff values of 0.3 to guide interpretations and modeling decisions. We conducted CFAs using the ‘lavaan’ package to build latent measurement models of internalizing and inflammation (Rosseel, 2012). Model fit was assessed with the χ^2^ goodness-of-fit statistic, confirmatory fit index ([CFI] Bentler, 1990), Tucker Lewis Index (TLI), standardized root mean square residual (SRMR), and root mean square error of approximation ([RMSEA] Steiger, 1990). In addition, Akaike information criterion (AIC) and Bayesian information criterion (BIC) were used to compare different models.

We used a series of hierarchical linear regressions to predict the factor scores of inflammation (Aim 1). In the first step, ACEs and the broad internalizing factor were used as separate main effect predictors. The interaction term with internalizing and ACEs was included in the second step. Johnson-Neyman analyses were used to probe and visualize the interaction. Sociodemographic covariates were included in the third step and health covariates were introduced in the fourth step. This was done to explore if the internalizing-ACEs interaction term predicted inflammation over-and-above the associations between covariates and inflammation.

Inclusion of health covariates in particular can diminish effect sizes between mood indices and inflammation (Appleton, Buka, Loucks, Gilman, & Kubzansky, 2013; Winter, Riordan, Conner, & Jose, 2021). This may be due to shared processes underlying inflammation and physical health. We performed follow-up analyses considering each health covariate individually. After observing how BMI eliminated the significant internalizing-ACEs effect, we repeated hierarchical regression models using BMI as the outcome variable instead of inflammation. Therefore, we tested if internalizing distress was functioning as a similar vulnerability with ACEs when predicting BMI.

Finally, we examined whether transdiagnostic internalizing or disorder-specific features moderated the association between ACEs and inflammation (Aim 2; i.e. broad comorbidity *v*. disorder-specific symptoms). Two simultaneous interaction terms were incorporated into the model: ACEs interacting with the broad internalizing factor scores and ACEs interacting with each unique residual component for a specific disorder. Only one disorder residual was included at a time. The neuroticism residual was conceptualized as measurement error and was not included. Neuroticism was included solely to improve our estimate of latent internalizing distress given the item content with relevance for all three disorder-specific domains in these models. For all study models, main and interaction effects were examined at *α* = 0.05.

## Results

### Latent measurement models

**Figure 1.**
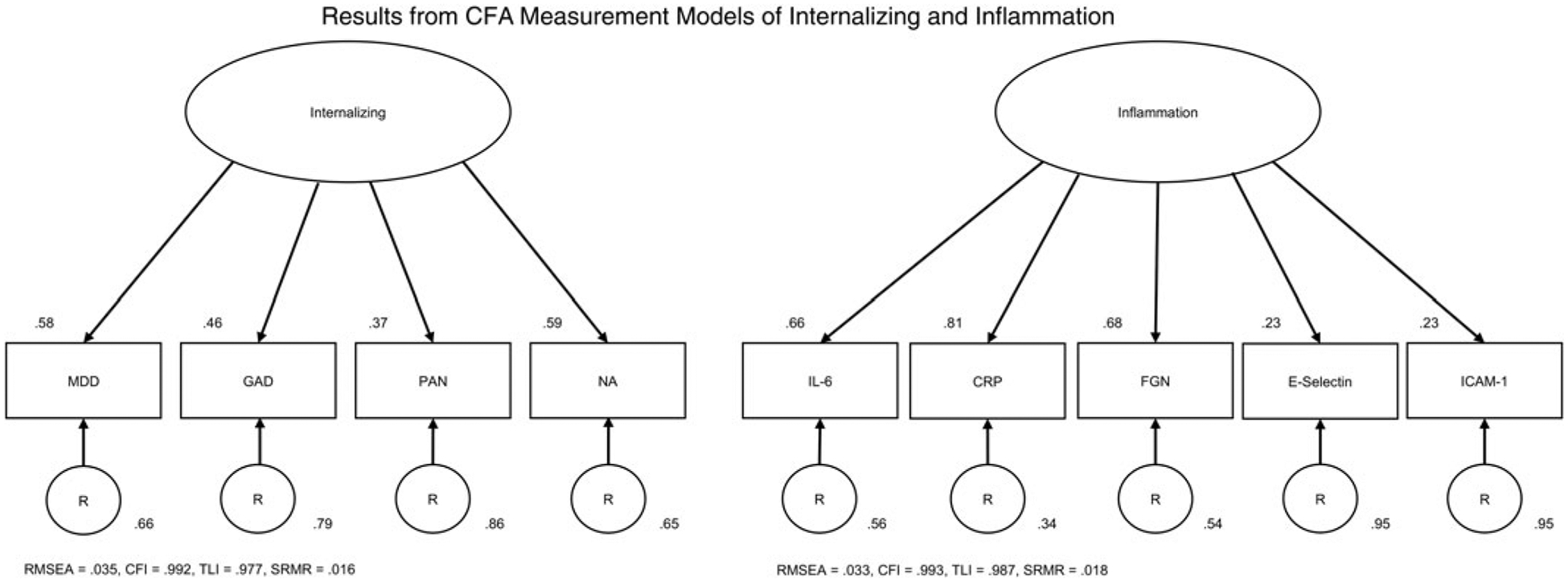
Results from CFA measurement models of internalizing and inflammation. Note: Arrows flowing from the latent factor to its indicators represent factor loadings. Arrows leading from each of the residuals to the indicators represent residual variance. Factor loadings and residual variances obtained using the completely standardized solution. Model fit indices are listed below each measurement model. MDD – major depressive disorder, GAD – generalized anxiety disorder, PAN – panic disorder, NA – neuroticism, IL-6 – interleukin 6, CRP – c-reactive protein, FGN – fibrinogen, ICAM-1 – intercellular adhesion molecule 1, RMSEA – root mean square error of approximation, CFI – comparative fit index, TLI – Tucker-Lewis index, SRMR – standardized root mean square residual.

All inflammation markers were positively correlated (mean *r* = 0.26, range [0.09–0.52]; Table 2). EFA supported either a one- or two-factor solution. The one-factor CFA model showed excellent fit (Fig. 1). A two-factor model was initially considered because markers E-Selectin and ICAM-1 had EFA loadings below 0.6 in the one-factor model, but CFA fit for the two-factor model (CFI = 0.99, TLI = 0.98, RMSEA = 0.04, SRMR = 0.02, AIC = 16 788, BIC = 16 845) did not meaningfully improve over the one-factor model (AIC = 16 788, BIC = 16 840). Therefore, the shared variance of the inflammatory indicators was extracted using a single inflammation factor.

All four internalizing distress measures were positively correlated (mean *r* = 0.24, range [0.13, 0.33]; Table 2). EFA results were consistent with the one-factor solution reported in past MIDUS analyses (Kim et al., 2021). The CFA model of internalizing exhibited excellent fit (Fig. 1). Both shared (transdiagnostic internalizing distress) and unique components (depression, anxiety, and panic) were extracted for subsequent analyses.

## Internalizing distress psychopathology and early adversity

**Figure 2.**
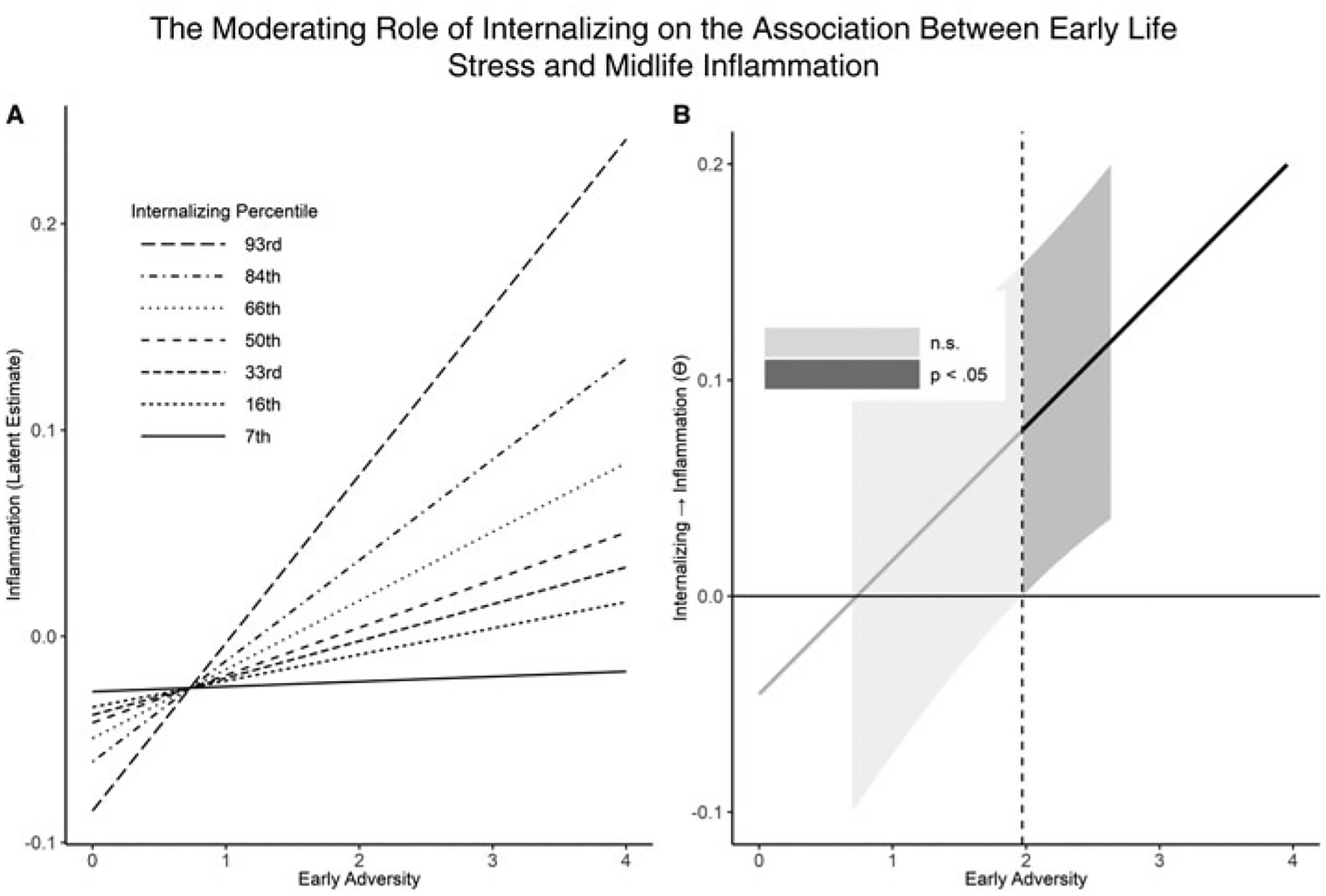
The moderating role of internalizing on the association between early life stress and midlife inflammation. Note: In Figure 2A, associations between early adversity and inflammation are depicted based on internalizing scores. Figure 2B presents a Johnson-Neyman plot illustrating the interaction and highlighting the threshold value of early adversity where a significant interaction emerges. n.s. – not significant.

We examined internalizing distress, ACEs, and their associations with inflammation (Table 3). For the first model step, internalizing (*β* = 0.083) and ACEs (*β* = 0.060) both independently predicted greater inflammation. With the second step, the interaction term between internalizing and ACEs was significantly associated with inflammation (*β* = 0.141); Fig. 2). Johnson-Neyman analyses revealed that a minimum of 2 ACEs was required before a significant positive association emerged between transdiagnostic internalizing distress and inflammation. Also, internalizing distress scores needed to be at the 53rd percentile before a positive association emerged between ACEs and inflammation. After adding sociodemographic variables as covariates in the third step, the interaction between internalizing and ACEs remained significant (*β* = 0.130).

When adding multiple health covariates simultaneously in the fourth step, the statistical interaction with ACEs and internalizing became non-significant (*β* = 0.070). We repeated the model by instead adding one health covariate at a time. We found that the interaction between ACEs and internalizing distress was only non-significant when BMI was included. Given the positive correlation between BMI and latent inflammation (*r* = 0.43), it is possible that BMI may reflect a similar biological process as the inflammation biomarkers. Subsequently, we examined if transdiagnostic internalizing distress and ACEs similarly predicted BMI. First, the internalizing factor and ACEs were used as main effect predictors for BMI (online Supplementary Table S1). Only ACEs exhibited a positive association with BMI (*b* = 0.435, *p* < 0.001, 95% CI [0.169–0.701], *β* = 0.096); internalizing distress was not significant (*b* = 0.325, *p* = 0.429, 95% CI [−0.481 to 1.13], *β* = 0.024). In the second model step, the ACEs-internalizing interaction term was significantly associated with BMI (*b* = 0.705, *p* = 0.004, 95% CI [0.220–1.189], *β* = 0.051). Johnson-Neyman analyses revealed that a minimum of 3 ACEs was required before a significant positive association emerged between the internalizing factor and BMI. Also, internalizing distress scores needed to be at the 39th percentile before a positive association between ACES and BMI emerged. Thus, we observed a similar moderation effect between transdiagnostic internalizing distress and ACEs when predicting BMI.

We also created two new ACEs subscales focused on abuse and neglect (see online Supplemental Materials). There was a significant interaction between abuse and latent internalizing (*b* = 0.094, *p* = 0.003, 95% CI [0.031–0.157], *β* = 0.137). Abuse predicted inflammation, but only when current internalizing was also elevated. In a separate model, neglect predicted inflammation as a main effect, but the interaction term with latent internalizing was not significant (*b* = 0.063, *p* = 0.053, 95% CI [−0.001 to 0.128], *β* = 0.101). Although the effect sizes are similar, abuse better explained the ACEs total score -by- internalizing moderation effect described above. Online Supplemental Tables S2–S4 summarize results comparing the broad internalizing factor score *v*. the disorder-specific residuals. The residual of depression did not interact with ACEs to predict inflammation (*b* = −0.010, *p* = 0.319, 95% CI [−0.048 to 0.016], *β* = −0.008), but the interaction between ACEs and broad internalizing remained significant (*b* = 0.077, *p* = 0.005, 95% CI [0.022–0.123], *β* = 0.173). Similarly, the anxiety residual did not significantly interact with ACEs to predict inflammation (*b* = 0.020, *p* = 0.281, 95% CI [−0.010 to 0.041], *β* = 0.015), whereas broad internalizing and ACEs remained significant (*b* = 0.062, *p* = 0.042, 95% CI [0.002–0.104], *β* = 0.140). Lastly, the panic residual did not significantly interact with ACEs to predict inflammation (*b* = 0.004, *p* = 0.754, 95% CI [−0.019 to 0.026], *β* = 0.001), but broad internalizing and ACEs maintained significance (*b* = 0.071, *p* = 0.012, 95% CI [0.014–0.108], *β* = 0.160). In summary, the transdiagnostic internalizing distress dimension was a better predictor of vulnerabilities to ACEs than the disorder-specific residuals of anxiety, depression, or panic.

## Discussion

Contrary to previous reports of a simple relationship between early adversity and midlife inflammation, our results revealed that this association depends on transdiagnostic internalizing distress. Within the MIDUS sample, heightened inflammation was best explained when ACEs were also combined with elevations on a broad factor score encompassing past 12-month internalizing symptomatology and trait neuroticism. We also observed that the disorder-specific residuals (MDD, GAD, panic) did not similarly moderate the adversity-inflammation effect.

These results point to the unique biological importance of internalizing comorbidities (DeYoung et al., 2023). Although chronic inflammation across the lifespan may be influenced by adversity, this effect may be at least somewhat conditional on the presence of processes shared across many forms of internalizing psychopathology. There are multiple possible causal sequences that could explain chronic inflammation in this sample. For example, the findings call to mind established models of resilience focused on the individual differences shaping adaptation following adversity (Luthar, Cicchetti, & Becker, 2000). Our trait-like rather than state-like index of internalizing and neuroticism may reflect stable vulnerabilities that amplified the long-term impact of adversity. A variety of transdiagnostic individual difference traits related to neuroticism are known to change the impact of stressors such as negative emotionality (Masten et al., 1999), rumination (Abela & Hankin, 2011), and maladaptive cognitive appraisal patterns (Hankin, Abramson, & Siler, 2001). Similar processes may have led to maladaptive stress responses among these MIDUS participants. Given the cross-sectional design, the extent to which features of internalizing distress were also present during childhood is unknown. It is possible adult internalizing distress is a continuation of the vulnerabilities present at the time of early stressors (Hilsman & Garber, 1995), which had long-term consequences on inflammation starting in childhood. It is also possible that current internalizing distress reflects ongoing impairments in stress regulation, which may be contributing to difficulties with managing responses to lingering psychological sequelae of past childhood adversity (e.g. intrusive reexperiencing; Ehlers and Steil, 1995). There is a need for additional prospective research to explore the longitudinal dynamics of these effects and determine whether pre-adversity internalizing distress is a true vulnerability for adversity-inflammation associations.

In contrast to a vulnerability hypothesis, a sensitization model highlights an alternative way ACEs together with internalizing may chronically exacerbate inflammation. Early adversity has a privileged role in shaping biological functioning, which can potentially sensitize reactivity to stressors later in life (Hostinar et al., 2015; Miller, Chen, & Parker, 2011). For instance, people who experienced childhood maltreatment can exhibit exacerbated acute inflammatory activity when exposed to present day stressors (Carpenter et al., 2010). Transdiagnostic internalizing psychopathology is a stressor because it disrupts role functioning and impairs quality of life. Consistent with this idea, Miller and Cole (2012) demonstrated that the onset of depressive symptoms among adult women was associated with more inflammation in those with childhood adversity. It is possible the immune response in people with ACEs has been sensitized to stress, which has led them to experience the impairment of internalizing psychopathology differently. Future studies of the inflammatory cascade may benefit from measurement approaches designed to tease apart the specific effects of current psychopathology *v*. the disability-related consequences of psychopathology.

For our second aim, we directly compared transdiagnostic and disorder-specific features of psychopathology. Inflammation was most closely associated with clinical symptomatology spanning across internalizing disorder measures. Therefore, the intercorrelations between internalizing symptoms and trait neuroticism were more than just a statistical phenomenon – these transdiagnostic associations best explained chronic inflammation following childhood adversity even when directly competing against symptoms specific to depression, anxiety, and panic. This finding provides a path toward synthesizing associations between various individual psychiatric diagnoses and inflammation (Duivis et al., 2013; Hoge et al., 2009; Michopoulos et al., 2017; Ogłodek et al., 2016; O’Donovan et al., 2010; Wagner et al., 2015). This is also thematically consistent with other studies of general health outcomes, which demonstrate stronger associations with broad internalizing than individual disorders (Eaton et al., 2013; Kim et al., 2021; van de Pavert, Sunderland, Luijten, Slade, & Teesson, 2017). By explicitly modeling comorbidities, our study demonstrates how transdiagnostic markers can help reveal stronger and more cohesive statistical associations with biological systems (DeYoung et al., 2023; Zald & Lahey, 2017).

There is value in considering why the moderation effect between ACEs and internalizing distress becomes non-significant after covarying for BMI. Elevated BMI is typically associated with higher adipose fatty tissue concentrations (de Heredia, Gómez-Martínez, & Marcos, 2012). Inflammatory markers such as IL-6 and CRP are synthesized in response to signaling molecules released through adipose tissue (Ouakinin, Barreira, & Gois, 2018). Moreover, research has established a bidirectional relationship between adipose tissue and clinical depression (Shelton & Miller, 2011). BMI may promote inflammation through similar underlying processes as internalizing psychopathology (e.g. activation of endocrine systems). Therefore, covarying for variables like BMI when predicting inflammation may remove meaningful variance (Slopen, Koenen, & Kubzansky, 2012). Notably, associations among internalizing conditions, inflammation, adverse childhood experiences, and BMI are frequently observed consistent with a shared pathophysiological process (Lamers, Milaneschi, de Jonge, Giltay, & Penninx, 2018). BMI may indirectly mediate the relationship between early adversity, adult internalizing, and adult inflammation (Palmos et al., 2019). Continuing examination of physiological contributors to chronic inflammation could improve our understanding of the underlying mechanistic pathways linking early adversity and internalizing distress.

This study has several limitations that point to important future directions of research. Although we used established methods for analyzing MIDUS inflammatory data (Elliot & Chapman, 2016; Friedman & Herd, 2010; Sheffler et al., 2019; Slopen et al., 2010), alternative approaches have been proposed. Representing inflammation as a latent construct involves measurement trade-offs. Aggregating multiple indicators together could mask genuine associations specific to particular inflammation indices (Moriarity & Alloy, 2021). It is also possible that replacing extreme inflammatory values removed some relevant information (Moriarity, 2022). Prior work has shown that baseline inflammation may lead to subsequent depressive symptoms (Valkanova, Ebmeier, & Allan, 2013), but the opposite has also been observed (Copeland, Shanahan, Worthman, Angold, & Costello, 2012; Glaus et al., 2014; Zainal & Newman, 2022). Due to the cross-sectional nature of the data, causality is difficult to determine, although the literature suggests some form of a bidirectional phenomenon. Recall of early adversity can be influenced by current distress (Martin-Wagar et al., 2024), which can result in substantially different associations between retrospective and prospective measures of ACEs (Colman et al., 2016; Hardt & Rutter, 2004). Studies of past adversity based solely on retrospective measures face challenges when trying to distinguish between current distress and ACEs. Race may also function as a proxy for adversity. Therefore, it is difficult to disentangle these relationships using cross-sectional designs. This was a motivating factor for using hierarchical models with and without covariates. Lastly, the CTQ, which contributed to our ACEs measure, has known limitations. These include a lack of cross-cultural considerations and variability in internal consistency across studies (Georgieva, Tomas, & Navarro-Pérez, 2021).

In conclusion, a latent factor reflecting past 12-month internalizing distress symptoms and trait neuroticism moderated the relationship between ACEs and midlife inflammation. Therefore, assessment of early adversity and internalizing distress in isolation appears to be insufficient – it was important to consider both together to capture the long-term inflammation effects more fully within the MIDUS sample. In addition, this transdiagnostic factor was a better predictor than disorder-specific facets of depression, anxiety, and panic. Given the growing research base validating transdiagnostic approaches for studying health outcomes, clinicians should be encouraged to also incorporate transdiagnostic symptom measures in their practice (Ruggero et al., 2019). Such assessments may reveal opportunities for intervention, which could shape the long-term physical health trajectories of patients with ACEs.

## Supplementary Material

Supplementals

## Figures and Tables

**Table 1.** Descriptive statistics of study sample

Measure	*n*	Mean (s.d.) or number (%)	Range
Internalizing domains			
Depressed affect	1255	0.58 (1.73)	0–7
Anxiety	1255	0.15 (0.96)	0–10
Panic	1255	0.31 (1.02)	0–6
Neuroticism	1249	2.05 (0.64)	1–4
Inflammatory markers			
IL-6 (pg/ml)	1243	3.04 (3.04)	0.16–23
CRP (μg/ml)	1235	3.02 (4.78)	0.02–61.7
FGN (mg/ml)	1235	348.92 (87.85)	45–857
E-selecting (ng/ml)	1242	43.39 (22.72)	0.09–178.05
ICAM-1 (ng/ml)	1242	288.55 (115.61)	44–1076.59
Demographics			
Age	1255	57.32 (11.55)	35–86
Gender (female)	1255	713 (57%)	0–1
Race/ethnicity :	1255		
White (non-Hispanic)		985 (78.5%)	
African American		215 (17.1%)	
And/or Black			
Native American		17 (1.4%)	
Asian		3 (0.2%)	
Other		31 (2.5%)	
Refused		4 (0.3%)	
Years of education	1252	7.47 (2.53)	1–12
Household income (ten thousands)	1213	41.5 (39.2)	0–200
Marital status :	1253		0–1
Married		811 (64.7%)	
Separated		34 (2.7%)	
Divorced		179 (14.3%)	
Widowed		77 (6.2%)	
Never married		152 (12.1%)	
Health covariates			
Body mass index	1215	28.52 (6.08)	14.23–61.43
Alcohol intake	1255	2.46 (1.55)	1–6
Exercise	1255	960 (76%)	0–1
Smoking status	1254	187 (15%)	0–1
Chronic conditions	1255	984 (78%)	0–1

*Note*. Descriptive statistics of study variables.

**Table 2.** Bivariate correlations amongst study variables

	1	2	3	4	5	6	7	8	9	10
1. MDD	-									
2. GAD	**0.27**	-								
3. PAN	**0.24**	**0.13**	-							
4. NA	**0.33**	**0.29**	**0.22**	-						
5. IL-6	0.04	**0.06**	0.04	−0.00	-					
6. CRP	**0.07**	**0.09**	**0.09**	0.05	**0.53**	-				
7. FGN	0.05	0.02	**0.06**	−0.01	**0.44**	**0.56**	-			
8. E-Selectin	0.03	−0.01	0.03	0.05	**0.20**	**0.17**	**0.15**	-		
9. ICAM-1	0.04	0.04	**0.06**	0.04	**0.18**	**0.18**	**0.14**	**0.09**	-	
10. ACEs	**0.23**	**0.20**	**0.17**	**0.27**	**0.07**	**0.09**	0.05	0.02	**0.08**	-

MDD, major depressive disorder; GAD, generalized anxiety disorder; PAN, panic disorder; NA, neuroticism; IL-6, interleukin 6; CRP, c- reactive protein; FGN, fibrinogen; ICAM-1, intercellular adhesion molecule; ACEs, adverse childhood experiences.

*Note*. Bolded coefficients *p* < 0.05. Internalizing and inflammatory measures are grouped separately in shaded boxes.

**Table 3.** Interactions between ACEs and internalizing on inflammation

Predictor	*b*	*β*	s.e.	*p*
Step 1				
**ACEs**	**0.036**	**0.083**	**0.013**	**<0.01**
**INT**	**0.079**	**0.060**	**0.039**	**<0.05**
Step 2				
**ACEs** × **INT**	**0.062**	**0.141**	**0.024**	<**0.01**
Step 3				
**ACEs** × **INT**	**0.056**	**0.130**	**0.023**	<**0.05**
Sociodemographic covariates				
Step 4				
ACEs × lNT	0.030	0.070	0.021	0.141
Sociodemographic + health covariates				

*Note*. Regression results using internalizing and ACEs as predictors. Bolded text indicates significant effect. ×: Examined for interaction. INT – latent internalizing. ACEs – adverse childhood experiences.
