# Supplementary material for: Early adversity and inflammation at midlife: the moderating role of internalizing psychopathology
Source: Psychol Med. Author manuscript; Available in PMC 2025 Jul 9. (PMC12238285; doi:10.1017/S0033291724002265)
Supplement: Supplementals [file NIHMS2049737-supplement-Supplementals.pdf]

# Early adversity and inflammation at midlife: The moderating role of internalizing psychopathology

***Supplemental Materials***

Michal et al.

**Table S1**

*Interaction Between Internalizing and ACEs on BMI*

| Predictor | *b* | *β* | *SE* | *p* |
|---|---|---|---|---|
| Step 1 | | | | |
| **ACEs** | **.435** | **.096** | **.136** | **< .001** |
| **INT** | .325 | .024 | .411 | .429 |
| Step 2 | | | | |
| **ACEs x INT** | **.705** | **.051** | **.247** | **.004** |

*Note. Regression results using internalizing and ACEs as predictors. Bolded text indicates significant effect. + : Examined for main effect. x : Examined for interaction. INT - Latent Internalizing. ACEs - Adverse Childhood Experiences.*

**Table S2**

*Exploring Interactions Between ACEs and Depression Residual on Inflammation*

| Predictor | *b* | *β* | *SE* | *p* | $R^2$ | $\Delta R^2$ |
|---|---|---|---|---|---|---|
| Step 1 | | | | | **.118** | - |
| INT | .076 [-.007, .160] | .058 | .043 | .074 | | |
| **ACEs** | **.032 [.011, .061]** | **.072** | **.013** | **.005** | | |
| Dep. Residual | .017 [-.043, .052] | .002 | .025 | .867 | | |
| Step 2 | | | | | **.142** | **.006** |
| **INT x ACEs** | **.077 [.022, .123]** | **.173** | **.025** | **.005** | | |
| ACEs x Dep. Residual | -.010 [-.048, .016] | -.008 | .016 | .319 | | |
| Step 3 | | | | | **.335** | **.092** |
| **INT x ACEs** | **.063 [.016, .113]** | **.143** | **.024** | **.009** | | |
| ACEs x Dep. Residual | -.008 [-.042, .018] | -.006 | .016 | .426 | | |
| Sociodemographic Covariates | | | | | | |
| Step 4 | | | | | **.525** | **.164** |
| INT x ACEs | .039 [.003, .092] | .087 | .022 | .084 | | |
| ACEs x Dep. Residual | -.018 [-.050, .004] | -.014 | .014 | .200 | | |
| Sociodemographics + Health Covariates | | | | | | |

*Note. Regression results using internalizing and ACEs as predictors and controlling for depression residual. Bolded text indicates significant effect. Bolded values in the rightmost column indicate that the test statistic resulting from the analysis of variance (ANOVA) comparing models is < 0.05. x : Examined for interaction. 95% Confidence interval of unstandardized beta reported in [brackets]. Dep. Residual : Depression Residual. INT - Latent Internalizing. ACEs - Adverse Childhood Experiences.*

**Table S3**

*Exploring Interactions Between ACEs and Anxiety Residual on Inflammation*

| Predictor | *b* | *β* | *SE* | *p* | $R^2$ | $\Delta R^2$ |
|---|---|---|---|---|---|---|
| Step 1 | | | | | .122 | - |
| INT | .066 [-.013, .147] | .051 | .041 | .102 | | |
| **ACEs** | **.032 [.011, .062]** | **.071** | **.013** | **.005** | | |
| Anx. Residual | .021 [-.018, .062] | .030 | .021 | .288 | | |
| Step 2 | | | | | .142 | **.005** |
| **INT x ACEs** | **.062 [.002, .104]** | **.140** | **.027** | **.042** | | |
| ACEs x Anx. Residual | .020 [-.012, .041] | .015 | .014 | .281 | | |
| Step 3 | | | | | .336 | **.093** |
| **INT x ACEs** | **.052 [.001, .099]** | **.116** | **.026** | **.044** | | |
| ACEs x Anx. Residual | .020 [-.010, .041] | .016 | .013 | .232 | | |
| Sociodemographic Covariates | | | | | | |
| Step 4 | | | | | .523 | **.161** |
| INT x ACEs | .012 [-.025, .064] | .026 | .024 | .381 | | |
| ACEs x Anx. Residual | .023 [-.008, .038] | .017 | .012 | .200 | | |
| Sociodemographics + Health Covariates | | | | | | |

*Note. Regression results using internalizing and ACEs as predictors and controlling for anxiety residual. Bolded text indicates significant effect. Bolded values in the rightmost column indicate that the test statistic resulting from the analysis of variance (ANOVA) comparing models is < 0.05. x : Examined for interaction. 95% Confidence interval of unstandardized beta reported in [brackets]. Anx. Residual : Anxiety Residual. INT - Latent Internalizing. ACEs - Adverse Childhood Experiences.*

**Table S4**

*Exploring Interactions Between ACEs and Panic Residual on Inflammation*

| Predictor | *b* | *β* | *SE* | *p* | $R^2$ | $\Delta R^2$ |
|---|---|---|---|---|---|---|
| Step 1 | | | | | .129 | - |
| INT | .065 [-.013, .143] | .050 | .040 | .105 | | |
| **ACEs** | **.031 [.010, .061]** | **.069** | **.013** | **.007** | | |
| Pan. Residual | .031 [-.002, .071] | .049 | .019 | .060 | | |
| Step 2 | | | | | .149 | **.006** |
| **INT x ACEs** | **.071 [.014, .108]** | **.160** | **.024** | **.012** | | |
| ACEs x Pan. Residual | .004 [-.019, .026] | .001 | .012 | .754 | | |
| Step 3 | | | | | .339 | **.093** |
| **INT x ACEs** | **.056 [.008, .098]** | **.127** | **.023** | **.022** | | |
| ACEs x Pan. Residual | .006 [-.013, .030] | .005 | .011 | .435 | | |
| Sociodemographic Covariates | | | | | | |
| Step 4 | | | | | .525 | **.161** |
| INT x ACEs | .026 [-.015, .068] | .059 | .021 | .210 | | |
| ACEs x Pan. Residual | .005 [-.010, .030] | .004 | .010 | .333 | | |
| Sociodemographics + Health Covariates | | | | | | |

*Note. Regression results using internalizing and ACEs as predictors and controlling for panic residual. Bolded text indicates significant effect. Bolded values in the rightmost column indicate that the test statistic resulting from the analysis of variance (ANOVA) comparing models is < 0.05. x : Examined for interaction. Examined for interaction. 95% Confidence interval of unstandardized beta reported in [brackets]. Pan. Residual : Panic Residual. INT - Latent Internalizing. ACEs - Adverse Childhood Experiences.*

**Comparing the Effects of Subgroupings of Adverse Childhood Experiences**
As a follow-up analysis, we created two new ACEs measures focused specifically on items related to abuse (risk to one's physical safety) or neglect (lack of adequate mental and social interaction) following the conceptual framework of earlier work (Sheridan & McLaughlin, 2014). For these supplemental analyses, we did not dichotomize the CTQ scales. The three scales of abuse (physical, sexual, and emotional) were z-transformed and then averaged to create the abuse dimension of ACEs. Subsequently, the neglect scales (physical and emotional) were z-transformed and averaged to make up the ACEs dimension of neglect. The two ACEs dimensions were analyzed separately in hierarchical regressions similar to the main paper looking at the interaction between the cumulative ACEs variable and latent internalizing.

The abuse dimension significantly predicted inflammation ($b$ = .084, β = .139, 95% CI [.043, .126], p < .001) whereas internalizing did not ($b$ = .053, β = .047, 95% CI [-.024, .131], p = .176). Next, when setting the two predictors to interact, a significant interaction between ACEs abuse dimension and internalizing emerged to predict inflammation ($b$ = .094, β = .137. 95% CI [.031, .157], p = .003). Johnson-Neyman plots revealed that when abuse z-scores were outside the range -.181-0.78, greater internalizing significantly predicted greater inflammation. Next, when including sociodemographic covariates into the model, the interaction term remained significant ($b$ = .078, β = .114, 95% CI [.018, .138], p = .011). Lastly, when adding the health covariates into the model, the interaction between ACEs abuse dimension and internalizing no longer significantly predicted inflammation ($b$ = .045, β = .065, 95% CI [-.010, .995], p = .112).

For the second model, ACEs neglect dimension significantly predicted inflammation (b = .059, β = .107, 95% CI [.022, .096], p = .002) whereas internalizing did not ($b$ = .073, β = .065, 95% CI [-.003, .149], p =.060). Next, when setting the two terms to interact, the term was not a significant predictor of inflammation ($b$ = .063, β = .101, 95% CI [-.001, .128], p = .053). Given the similar interaction effect size in this model compared with the first abuse model, we looked at Johnson-Neyman plots. Plots revealed that in a similar manner to abuse, when neglect is within 0.50-80.21, internalizing significantly predicts inflammation in the positive direction. Afterwards, we included sociodemographic covariates into the model and found that the interaction term's prediction on inflammation was attenuated to be non-significant ($b$ = .057, β = .091, 95% CI [-.005, .119], p = .070). Lastly, we added health covariates into the model and found similar results ($b$ = .034, β = .053, 95% CI [-.023, .090], p = .241).

These findings suggest that experiences of abuse and neglect during childhood may not result in the same immune alterations later in life in context of concurrent emotional distress. While both abuse and neglect were predictive of inflammation, their interactions with internalizing symptoms varied in strength. While different types of childhood adversity may independently contribute to immune dysregulation, the dependency of these effects with respect to current internalizing psychopathology might vary. It is possible that experiences of abuse are associated with acute stress activation to a greater degree than neglect, and the abuse dimension may be particularly impactful on immune function when combined with internalizing psychopathology during midlife. However, the magnitude of the effect differences between abuse and neglect were small. Further research with more comprehensive assessments of types of childhood adverse experiences is needed.

**Table S5**

*Exploring Interactions Between Internalizing and Abuse Dimension on Inflammation*

| Predictor | *b* | *β* | *SE* | *p* | $R^2$ | $\Delta R^2$ |
|---|---|---|---|---|---|---|
| Step 1 | | | | | | |
| **Abuse** | **.084** | **.139** | **.021** | **<0.01** | | |
| INT | .053 | .047 | .038 | .176 | .018 | |
| Step 2 | | | | | | |
| **Abuse x INT** | **.094** | **.137** | **.032** | **.003** | .024 | .006 |
| Step 3 | | | | | | |
| **Abuse x INT** | **.078** | **.114** | **.031** | **.011** | .107 | .083 |
| Sociodemographic Covariates | | | | | | |
| Step 4 | | | | | | |
| Abuse x INT | .045 | .065 | .028 | .112 | .265 | .158 |
| Sociodemographics + Health Covariates | | | | | | |

*Note. Regression results using internalizing and ACEs as predictors. Bolded text indicates significant effect. + : Examined for main effect. x : Examined for interaction. INT - Latent Internalizing. ACEs - Adverse Childhood Experiences.*

**Table S6**

*Exploring Interactions Between Internalizing and Neglect Dimension on Inflammation*

| Predictor | *b* | *β* | *SE* | *p* | $R^2$ | $\Delta R^2$ |
|---|---|---|---|---|---|---|
| Step 1 | | | | | | |
| **Abuse** | **.059** | **.107** | **.019** | **.002** | | |
| INT | .073 | .065 | .039 | .060 | .013 | |
| Step 2 | | | | | | |
| Abuse x INT | .063 | .101 | .033 | .053 | .015 | .002 |
| Step 3 | | | | | | |
| Abuse x INT | .057 | .091 | .031 | .070 | .100 | .085 |
| Sociodemographic Covariates | | | | | | |
| Step 4 | | | | | | |
| Abuse x INT | .034 | .053 | .029 | .241 | .265 | .165 |
| Sociodemographic + Health Covariates | | | | | | |

*Note. Regression results using internalizing and ACEs as predictors. Bolded text indicates significant effect. + : Examined for main effect. x : Examined for interaction. INT - Latent Internalizing. ACEs - Adverse Childhood Experiences.*

**Exploring a Socioeconomic Status Composite**

We created a composite proxy of socioeconomic status (SES) following Elliot and colleagues (Elliot et al., 2017). Specifically, highest level of education (ranged from 1 = no school/grade school to 12 =Ph.D./M.D./professional degree), total household income (continuous measure of total family income in the past 12 months ), and occupational prestige (MIDUS item that ranked participants' occupation based on the Duncan SEI (Hauser & Warren, 1997) were all z-transformed and combined in an average. We modeled a regression similar to Elliot in that a neuroticism proxy was set to interact with the SES composite to predict inflammation. We differed by using the latent inflammation composite as the dependent variable rather than predicting individual markers IL-6 and CRP separately. The interaction between SES and neuroticism was similar to Elliot's results ($b = -.042$, $\beta = -.131$, 95% CI [-.086, .001], $p = 0.056$). Johnson-Neyman plots revealed that the trend of the interaction remained consistent with Elliot's in that less neuroticism was associated with lower inflammation at lower levels of SES, though our results were not significant. The association is likely nuanced and because the original analyses modeled individual markers as the dependent variable, it is possible that type I errors were more likely and therefore resulted in slightly varied results.

**Table S7**

*Interaction Between SES Composite and Neuroticism Using Inflammation as an Outcome*

| Predictor | *b* | *β* | *SE* | *p* |
|---|---|---|---|---|
| Step 1 | | | | |
| **SES** | **-.099** | **-.153** | **.022** | **< .001** |
| NEURO | -.010 | -.020 | .017 | .574 |
| Step 2 | | | | |
| SES x NEURO | -.042 | -.131 | .022 | .057 |

*Note. Regression results using NEURO and SES composite as predictors. Bolded text indicates significant effect. + : Examined for main effect. x : Examined for interaction. NEURO - Neuroticism. SES – Socioeconomic Status.*
